# Genetic characterization of human coxsackievirus A6 variants associated with atypical hand, foot and mouth disease: a potential role of recombination in emergence and pathogenicity

**DOI:** 10.1099/vir.0.000062

**Published:** 2015-05

**Authors:** Eleanor Gaunt, Heli Harvala, Riikka Österback, Vattipally B. Sreenu, Emma Thomson, Matti Waris, Peter Simmonds

**Affiliations:** ^1^​ Roslin Institute, University of Edinburgh, Easter Bush, Edinburgh EH25 9RG, UK; ^2^​ Specialist Virology Laboratory, Royal Infirmary of Edinburgh, Edinburgh, UK; ^3^​ Department of Virology, University of Turku, 20520 Turku, Finland; ^4^​ MRC University of Glasgow Centre for Virus Research, 8 Church Street, Glasgow G11 5JR, UK

## Abstract

Human coxsackievirus A6 (CVA6) is an enterically transmitted enterovirus. Until recently, CVA6 infections were considered as being of minor clinical significance, and only rarely aetiologically linked with hand, foot and mouth disease (HFMD) associated with other species A enteroviruses (particularly EV71 and CVA16). From 2008 onwards, however, CVA6 infections have been associated with several outbreaks worldwide of atypical HFMD (aHFMD) accompanied by a varicelliform rash. We recently reported CVA6-associated eczema herpeticum occurring predominantly in children and young adults in Edinburgh in January and February 2014. To investigate genetic determinants of novel clinical phenotypes of CVA6, we genetically characterized and analysed CVA6 variants associated with eczema herpeticum in Edinburgh in 2014 and those with aHFMD in CAV isolates collected from 2008. A total of eight recombinant forms (RFs) have circulated worldwide over the past 10 years, with the particularly recent appearance of RF-H associated with eczema herpeticum cases in Edinburgh in 2014. Comparison of phylogenies and divergence of complete genome sequences of CVA6 identified recombination breakpoints in 2A–2C, within VP3, and between 5′ untranslated region and VP1. A Bayesian temporal reconstruction of CVA6 evolution since 2004 provided estimates of dates and the actual recombination events that generated more recently appearing recombination groups (RF-E, -F, -G and -H). Associations were observed between recombination groups and clinical presentations of herpangina, aHFMD and eczema herpeticum, but not with VP1 or other structural genes. These observations provided evidence that NS gene regions may potentially contribute to clinical phenotypes and outcomes of CVA6 infection.

## Introduction

*Human coxsackievirus A6* (CVA6) is a member of the genus *Enterovirus* in the family *Picornaviridae*, a group of primarily enteric RNA viruses that cause a wide range of diseases in humans and other mammals (Knowles *et al.*, 2012). There are currently >100 recognized human enterovirus serotypes, classified into four species (EV-A to -D). EV-A species are widely distributed in human and non-human primate populations, and those infecting humans currently comprise a total of 20 (sero)types. Of these, EV71 and CAV16 are the most closely linked to hand, foot and mouth disease (HFMD), occasionally leading to fatal meningoencephalitis (Solomon *et al.*, 2010).

By contrast, although widely circulating in the community, CVA6 has rarely attracted clinical attention as infections are typically mild or asymptomatic. However, a change in its clinical phenotype was reported in 2008, following the discovery of a large outbreak of HFMD associated with CVA6 in Finland (Österback *et al.*, 2009). Since then, numerous case and surveillance reports have documented substantial increases in HFMD incidence in Europe, Asia and the USA, in each case strongly associated with CVA6 infections, and eclipsing EV71 and CAV16 as causative agents (Chen *et al.*, 2012; Chung *et al.*, 2013; Di *et al.*, 2014; Feder *et al.*, 2014; Fujimoto *et al.*, 2012; Huang *et al.*, 2013; Kar *et al.*, 2013; Kobayashi *et al.*, 2013; Lo *et al.*, 2011; Lu *et al.*, 2012; Mathes *et al.*, 2013; Montes *et al.*, 2013; Puenpa *et al.*, 2014; Sinclair *et al.*, 2014; Wei *et al.*, 2011; Yasui *et al.*, 2013). Moreover, several of these studies recorded differences in symptomatology from HFMD disease arising from EV71 and CVA16 infections; CVA6-associated atypical HFMD (aHFMD) is frequently associated with crusted lesions, an eczema-form rash affecting the arms, trunk, buttocks and legs, onychomadesis (nail loss), and a greater disease severity than HFMD associated with other EV-A serotypes (Chung *et al.*, 2013; Mathes *et al.*, 2013; Yasui *et al.*, 2013). One study from Thailand also described frequent associations with high fever (Puenpa *et al.*, 2014).

We recently reported a cluster of clinically presenting CVA6 infections in January and February 2014 in Edinburgh (Sinclair *et al.*, 2014). In these, hospitalized cases presented with a rash diagnosed as eczema herpeticum by dermatologists and initially leading to clinical diagnoses of severe, disseminated herpes simplex virus infections. The erythematous papular rash involved the face, trunk and limbs, and was followed by the appearance of vesicles and bullae. Sequence analysis of the VP1 gene demonstrated that most variants associated with the outbreak were genetically distinct from other previously characterized CVA6 strains, including those associated with all previous outbreaks of aHFMD since 2008 and all earlier isolates.

The underlying reasons for these changes in clinical phenotype of CVA6 are unknown. Host factors, such as herd immunity and age of infection, are known to influence outcomes of enterovirus infections, but the evidence for its rapid emergence worldwide argues strongly against population-wide changes in host disease susceptibility to CVA6. To investigate whether virus-specific factors were involved, we genetically analysed CVA6 variants associated with the recent Edinburgh aHFMD cases and those associated with the earlier outbreak in Finland in 2008 in multiple gene regions. In addition, complete genome sequences were obtained from 15 CVA6 strains. Two novel recombinant forms (RFs) of CVA6, defined by phylogeny relationships in the non-structural (NS) gene region, were identified that were associated specifically with the recent aHFMD cases in Edinburgh (RF-H) and previous outbreaks (RF-A). Although we lack the experimental models to precisely identify determinants of virus pathogenicity in human enterovirus infections, these observations provide evidence for a role of virus recombination in the emergence of new CVA6 strains with altered clinical phenotypes.

## Results

### CAV-6 variants associated with aHFMD and eczema herpeticum

**Fig. 1.**
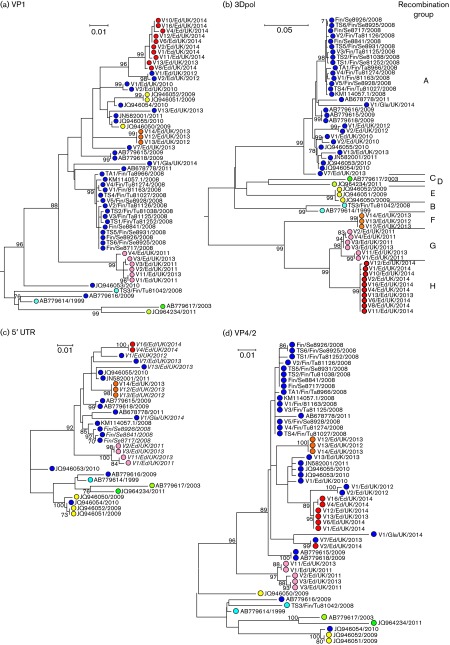
Phylogeny of CVA6 variants in different genome regions. Maximum-likelihood tree of (a) VP1 [positions 2430–3267 numbered using the reference CVA6 sequence, Gdula (GenBank accession number AY421764)], (b) 3Dpol (positions 6245–6988), (c) 5′ UTR (positions 24–752) and (d) VP4/partial VP2 (positions 753–1187). Trees were reconstructed using optimal substitution models: Kimura two-parameter (K2P)+invariant sites (I) for VP1; K2P+I+gamma distribution (Γ) for 3Dpol; K2P+I for 5′ UTR and K2P+Γ for VP4/2. Trees were rooted with the prototype sequence, GenBank accession number AY421764 (not shown). Bootstrap resampling (100 replicates) was used to determine robustness of groupings; values of ≥70 % shown. Sequences were coloured according to their recombination group assignments based on 3Dpol phylogeny.

All 10 of the patients with eczema herpeticum presenting in 2014 in Edinburgh were infected with CVA6 variants that were phylogenetically distinct from other previously characterized variants of that serotype in the VP1 region (Sinclair *et al.*, 2014). An extended comparison in the VP1 region was performed using further samples collected before the outbreak in Edinburgh (*n* = 16) and several CVA6 variants associated with the outbreak of aHFMD in Finland in 2008 (*n* = 15; Fig. 1a). Despite the inclusion of these additional sequences, the separate grouping of the viruses detected in the Edinburgh cohort remained and was also distinct from all CVA6 variants associated with aHFMD elsewhere (Finland, Taiwan, Japan and China; Fig. 1a). Variants from the USA, Spain and France were shown previously to be distinct (Sinclair *et al.*, 2014).

To investigate sequence relationships in other parts of the genome, nucleotide sequences were obtained from the 3Dpol region, representing the coding region furthest away from VP1, as well as VP4 and the 5′ UTR. The dataset was supplemented by 15 (near-)complete genome sequences of CVA6 variants from the UK (*n* = 12) and Finland (*n* = 3). In the 3Dpol region, sequences fell into eight primary clades, each with 100 % bootstrap support (Fig. 1b). Following the practice of previous analyses of recombination in enteroviruses (McWilliam Leitch *et al.*, 2009, 2012), variants were assigned into RF-A to -H based on 3Dpol phylogeny (and coloured from blue to red; RF-I was assigned to the outgroup sequence of the prototype CVA6 strain isolated in 1949). A large number of phylogeny differences were apparent between this tree and the tree from VP1.

**Fig. 2.**
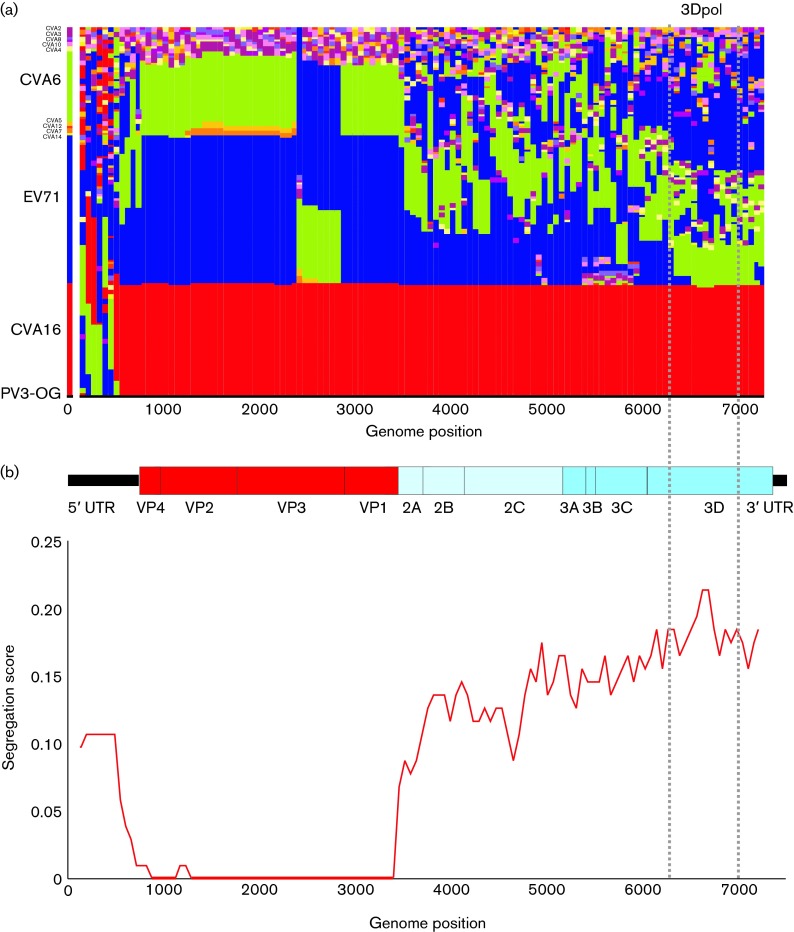
Tree positions (TreeOrder scan) of species A serotypes across the genome. (a) Tree positions represented on the *x*-axis of available complete genome sequences of EV-A, including CVA6 genomes obtained in the current study. Sequences of different EV types are coloured as labelled on the left-hand *y*-axis. The analysis excluded the more divergent EV76, EV89–91 and other simian-derived types, with the trees rooted using the poliovirus type 3 reference sequence, Leon (GenBank accession number K01392, labelled PV3-OG). Trees were reconstructed from sequential 300-base fragments of the EV-A sequence alignment, incrementing by 30 bases between trees. Groupings are based on clades showing ≥70 % bootstrap support. (b) Correspondence between the phylogeny of sequential fragments through the enterovirus genome with their type assignments, scored from 0 (complete concordance) to 1 (no association) as described previously (Simmonds & Welch, 2006). An enterovirus genome diagram drawn to scale is included to indicate positions where segregation changes occurred. Sequence positions are numbered relative to the poliovirus type 3 Leon sequence outgroup (GenBank accession number K01392).

3Dpol region sequences from the Edinburgh 2014 cohort fell into a clade (RF-H) distinct from all other CVA6 strains characterized to date, including variants collected from Edinburgh before the outbreak and variants associated with previous aHFMD outbreaks in Finland, Japan and Taiwan. As previously observed for other enterovirus serotypes (McWilliam Leitch *et al.*, 2009, 2010, 2012), CVA6 RFs possessed 3Dpol sequences that were interspersed with corresponding sequences from other EV-A serotypes (Fig. 2 and Fig. S1, available in the online Supplementary Material), which contrasts with their consistent grouping by serotype in the structural gene region. Despite this, none of the eight CAV recombination groups identified in the current study shared 3Dpol region sequences with other species A serotypes sequenced in this region.

The occurrence of recombination in the NS genome region was demonstrated by a marked rise in segregation score (indicating non-concordance of phylogeny positions with group assignments) at the end of VP1 and towards the start of VP4 (Fig. 2b). Intriguingly, the only exception was CAV16 which, despite extensive sampling and availability of multiple complete genome sequences, remained almost entirely monophyletic throughout the genome apart from the 5′ end of the 5′ UTR (Fig. 2a). A bootstrap-supported clade containing all but one CAV16 sequence [the prototype sequence with GenBank accession number AY421764 (Gdula isolate)] was clearly evident in the 3Dpol region distinct from all other variants (Fig. S1). In contrast to the NS gene region, the phylogeny of VP4/2 and the 5′ UTR was broadly consistent with VP1 (Fig. 1c, d). The main difference was between the grouping of RF-E with members of RF-A and -H (both associated with HFMD) in VP1, but its outlier position in 5′ UTR and VP4/2.

**Fig. 3.**
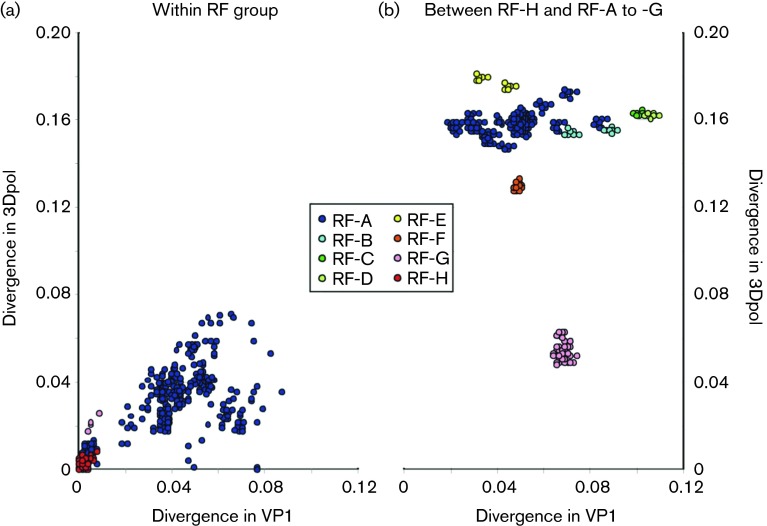
Comparison of pairwise distances in VP1 and 3Dpol between and within recombination groups. Comparison of divergence between VP1 and 3Dpol regions (a) within recombination groups, and (b) between RF-H and other recombination groups. Axes depict nucleotide sequence divergence (uncorrected *p* distances) in the two genome regions.

The existence of recombination between genome regions was also demonstrated through comparison of pairwise distances between sequences in VP1 and those in 3Dpol (*x*- and *y*-axes in Fig. 3, respectively) as previously performed for EV71 (McWilliam Leitch *et al.*, 2009). Within the RF-A group, most sequence distances in the two genome regions were comparable and a plot of pairwise distances fell largely on a straight line, reflecting similar degrees of divergence in the two genome regions (Fig. 3a). There were, however, a number of exceptions, with some variants being less divergent in 3Dpol than would be expected from their degree of sequence divergence in VP1. These may represent within-group recombinants, in which a number of RF-A variants recombined with each other, acquiring less divergent NS gene sequences than their sequences in VP1.

Further comparison of VP1 and 3Dpol region sequences between RF-A and -H, and between RF-H and other recombination groups, demonstrated that each possessed far more divergent 3Dpol sequences than would be expected based on VP1 pairwise distances (Fig. 3b). This was consistent with numerous acquisitions of NS gene regions from other EV-A species. The only exception was between RF-H and -G, where mean sequence divergence in VP1 (0.069) was comparable with that in 3Dpol (0.058), implying that these two genome regions have co-diverged for a considerable time. The reason why these are classified as different recombination groups is explained below.

### Reconstructing the timescale of specific recombination events in CVA6

**Fig. 4.**
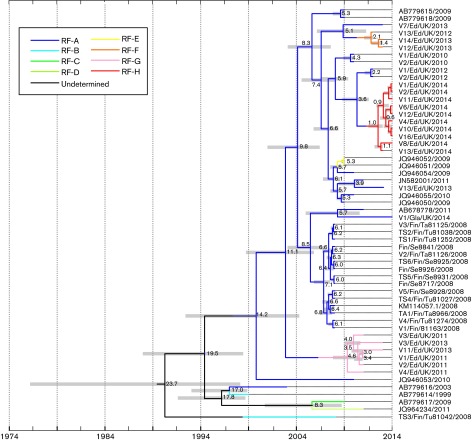
Inferred timescale for the recent evolution of CVA6. Temporal reconstruction of recombination events in CVA6 using a time-correlated MCMC phylogeny reconstruction of VP1 sequences using the prototype sequence, GenBank accession number AY421764, as an outgroup (not shown). Recombination groups in each lineage are indicated by branch colours. The tree is plotted on a linear timescale, with nodes labelled with inferred dates of lineage splits (in years before present). Grey bars show 95 % highest posterior density intervals for node date estimates.

To investigate when recombination groups of CVA6 first appeared, sequences from the VP1 region were analysed by Markov chain Monte Carlo (MCMC) analysis using sample dates to reconstruct a temporal phylogeny (Fig. 4). The substitution rate of CVA6 VP1 sequences was estimated at 4.2×10^−3^ substitutions site^−1^ year^−1^ (2.8–5.8×10^−3^ highest posterior density interval), comparable with that of EV71 and other species A serotypes in previous analyses (Lukashev *et al.*, 2014; McWilliam Leitch *et al.*, 2009). Substitution rates were relatively uniformly distributed amongst different lineages in the VP1 tree (Fig. S2).

In the more recent evolution of CVA6, approximate dates of when recombination events must have occurred could be inferred from the temporal phylogeny (Fig. 4), in which the recombination groups of individual lineages were inferred in a reconstruction in which the fewest number of recombination events occurred (indicated by line colour). Whilst the small number of CVA6 variants isolated before 2008 precluded detailed reconstruction of earlier recombination events, the reconstruction nevertheless indicated that RF-A had a relatively long evolutionary history, with the main clade (excluding the earliest isolate dated from 2003; GenBank accession number AB779616) originally emerging between 1994 and 2000. Several recombination groups (RF-E, -F, -G and -H) subsequently emerged within this clade, presumably the results of separate recombination events with other species A serotypes over the past 10 years. The recombination events that generated RF-E and -F could be dated to ~3–5 years ago. The appearance of RF-H (or at least the variants found in Edinburgh) was the most recent (2.5 years), consistent with the limited sequence heterogeneity between VP1 sequences in this RF.

The deeper evolutionary history of CVA6 was more difficult to reconstruct because of very limited sampling and a paucity of characterized, more divergent CVA6 variants. There were indeed many similarly parsimonious recombination histories to account for the existence of RF-B to -D and the presence of the 2003 outlier RF-A variant, GenBank accession number AB779616 (which may conceivably have originated through a parallel recombination event). For these reasons, lineages reconstructed before the origin of the main RF-A clade were designated ‘Undetermined’.

### Mapping recombination breakpoints

**Fig. 5.**
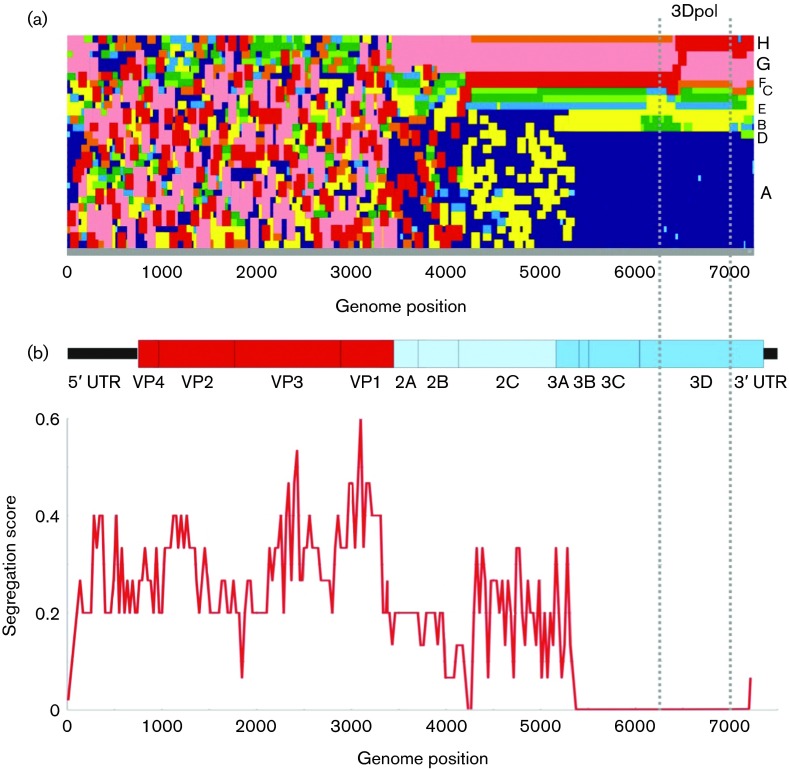
TreeOrder scan of CVA6 complete genome sequences. (a) Tree positions represented on the *y*-axis of CVA6 genomes with different recombination groups coloured as labelled on the right-hand *y*-axis. Trees were constructed from sequential 300-base fragments of the CVA6 sequence alignment, incrementing by 30 bases between trees. Groupings are based on clades showing ≥70 % bootstrap support. (b) Segregation scores (see legend to Fig. 2) for sequences classified by recombination group. Sequence positions are numbered relative to the poliovirus outgroup (GenBank accession number K01392).

A conceptual problem with determining where recombination occurred in the evolution of enterovirus RFs is the lack of any reference, non-recombinant sequences with which to compare them. The past evolution of EV-A was modular, and multiple occurrences of recombination were evident from numerous incompatibilities in structural and NS gene phylogenies in all extant variants (Fig. 1a, b). Consequently, there were no non-recombinant sequences of CVA6 with which to map breakpoints in RF-H. However, a broad guide as to where recombination typically occurs is provided by the TreeOrder scan of CVA6 sequences (Fig. 5a), in which sequences were colour coded by recombination group (RF-A to -H) and their tree positions recorded across the genome.

The analysis was based on available CVA6 complete genome sequences and those of representative sequences from each of the recombination groups represented by samples from Edinburgh and Finland characterized in the current study (RF-A, -F, -G and -H). Sequences throughout 3B, 3C and 3Dpol (including the fragment used for RF assignments; shown between vertical dashed lines) consistently grouped by their recombination groups assignment with zero segregation scores (Fig. 5b). There were, however, some minor changes in tree order between clades in this region. Upstream, RF-A and -E disintegrated in the 3A region with RF-E variants becoming embedded within RF-A, followed by several other recombination groups around the 2B/2C junction.

**Fig. 6.**
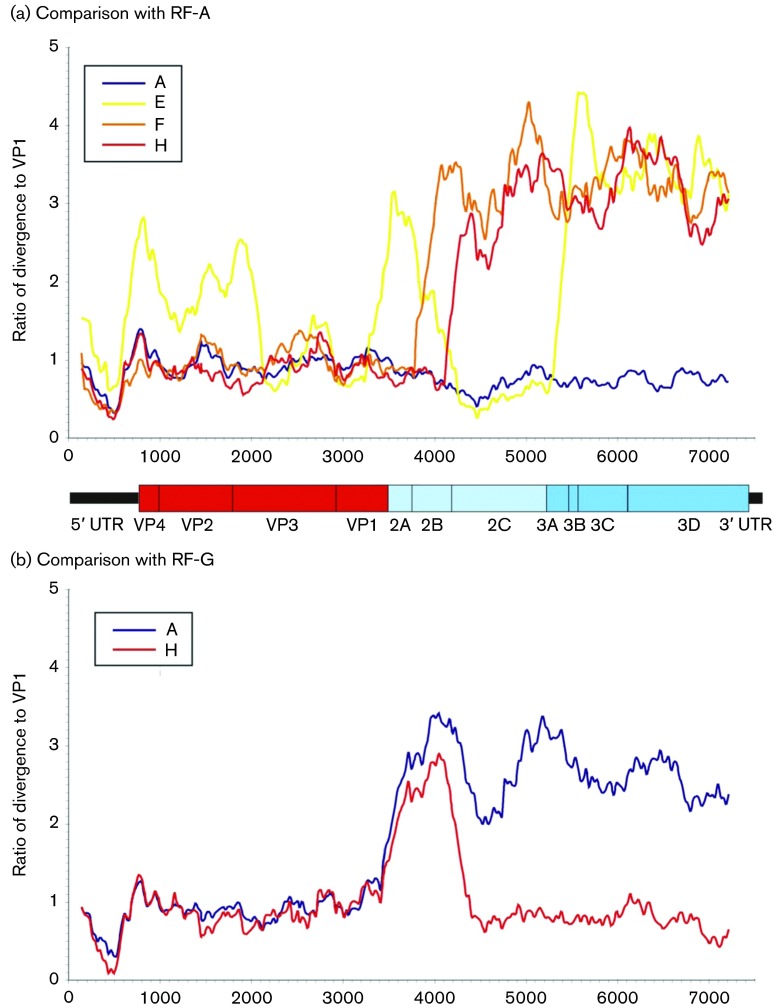
Divergence of RF-A and -G from other recombination groups across the CVA6 genome. Scan of nucleotide sequence divergence (a) between RF-A and descendant recombination groups (RF-E, -F and -H), and (b) between RF-G with RF-A and -H. Plotlines show ratios of divergence (*y*-axis) in different genome positions (*x*-axis) to divergence in the VP1 gene (positions 2441–3355). Distances were calculated between sequential 300-base fragments of the CVA6 alignment incrementing by 30 bases between data points.

As an alternative way to visualize sequence relationships, sequence alignments were scanned to identify regions of the genome where sequence divergence became greater than in VP1. This analysis method was developed from the results of the dot-plot in which divergence in VP1 and 3Dpol was compared (Fig. 5b). This demonstrated ~3.5 times greater divergence between RF-H and other recombination groups in 3Dpol than in VP1, indicative of recombination. This analysis was extended by comparing ratios of VP1/3Dpol divergence in successive fragments across the genome to identify where changes in ratios occurred and thus identify recombination breakpoints (Fig. 6). In the case RF-E, -F, -G and -H, temporal reconstruction of VP1 sequences predicted that RF-A was ancestral to them (Fig. 4) and that they likely originated through separate recombination events. Divergence scans comparing RF-A with RF-F and -H confirmed this hypothesis (Fig. 6a), with divergence ratios close to 1 throughout the 5′ UTR, structural gene region and 2A, but steep increases in divergence relative to that of VP1 around positions 3900 and 4400 (both within 2B or at junctions with 2A and 2C). These localize likely recombination events with other (uncharacterized) EV-A variants at these genome positions. Sequence relationships with RF-E were more complex, indicative of multiple recombination events. Sequences between RF-A and -E were shared in VP3, VP1 and 2C, whilst the 5′ UTR, VP4 and VP2 were divergent. Further recombination events were predicted to yield divergent 2A and 2B genes, and a breakpoint within 3A was also apparent, which resulted in a high ratio for the rest of the genome downstream of this point. The high ratios observed in VP4/2 and the 5′ UTR were reflected in their outlier positions on phylogenetic comparisons from RF-A sequences (Fig. 1c, d).

A complex relationship between RF-G with RF-A and -H was also identified using this analysis (Fig. 6b). Comparing RF-G with RF-A and -H revealed a breakpoint at around position 3500 (within 2A), but in the case of RF-H, ratios returned to ~1 beyond position 4200 (2B/2C junction). Separate blast analysis of the 300-base segment within this inserted region of RF-G (positions 3850–4150) failed to identify any closely related sequences amongst other species A serotypes (minimum divergence of 15 %; data not shown). RF-G is therefore effectively a divergent version of RF-H throughout most of the genome (as indicated in the dot-plot), but with an insertion of a novel, NS gene region between VP1 and 3Dpol.

### Association of recombination group with clinical presentations

The current study obtained complete genome sequences and paired VP1/3Dpol sequences from a large number of CVA6 variants from Edinburgh and Finland, allowing associations between recombination group and clinical presentations to be compared. Our search of GenBank and PubMed databases identified four other studies from Finland (Österback *et al.*, 2014), Taiwan (Chen *et al.*, 2012; Chung *et al.*, 2013) and Japan (Fujimoto *et al.*, 2012) in which nine complete genome sequences were obtained from CVA6 infections with clinical descriptions (Table 1). Although comparing clinical descriptions from different sources was problematic, it was possible to broadly divide presentations into three categories: (1) herpangina and other non-HFMD presentations; (2) aHFMD with lesions extending to the hands, feet and perioral area, often clinically resembling chickenpox and being occasionally associated with onychomadesis (nail loss); and (3) extensive vesicular rash on the arms, legs and trunk, clinically resembling either eczematous dermatitis or eczema herpeticum (also termed eczema coxsackium; Mathes *et al.*, 2013) and occasionally being associated with onychomadesis.

**Table 1.** Association of recombination forms with clinical presentation See main text for definitions of the clinical presentation groups.

Recombination group	Herpangina, other	AHFMD	Ecezema herpeticum
RF-A	1	23 (4)*	2 (1)
RF-B	1 (1)	1	0
RF-C	0	0	0
RF-D	0	0	0
RF-E	3 (3)	0	0
RF-F	0	1	0
RF-G	0	4	1
RF-H	0	2	6

* Additional examples within the total derived from published descriptions shown in parentheses.

Although numbers were small, RF-A, along with RF-G, showed a strong association with aHFMD that contrasted with presentations of herpangina of RF-B and -E. Of note were the six cases of eczema herpeticum in those infected with RF-H – a presentation unusual or absent for other recombination groups. However, whilst we noted these associations, it was not possible to perform formal statistical significance testing of differences in clinical outcomes between RFs because infections were not phylogenetically or epidemiologically independent.

## Discussion

### Recombination in CVA6

The variability in phylogeny relationships between CVA6 variants in different genome regions (Figs 1, 3 and 4) is indicative of the occurrence of multiple recombination events between the structural gene region and other parts of the genome. These observations are consistent with a model of the modular evolution of enteroviruses, in which the diversification of the capsid gene, which determines serological properties (Lukashev, 2005; Oberste *et al.*, 2004a; Santti *et al.*, 1999), is largely uncoupled from that of NS genes and the 5′ UTR (Lukashev, 2005; Lukashev *et al.*, 2005; McWilliam Leitch *et al.*, 2009; Simmonds, 2006). Individual serotypes, such as echoviruses E30 and E11 in EV-B species and EV71 in EV-A species, have been documented to periodically exchange entire or near-complete NS gene regions with other enteroviruses; in E30, a regular succession of different recombination groups appeared throughout Northern and Western Europe over a 10-year study period with particularly dramatic changes in recombination group frequencies coinciding with peaks in its 4–5 year incidence cycle (Bailly *et al.*, 2009; McWilliam Leitch *et al.*, 2009). Other serotypes, such as E6, E9 and E11 echoviruses, showed successions of recombination groups, but with different dynamics and periodicities (Cabrerizo *et al.*, 2014; McWilliam Leitch *et al.*, 2010). In our investigation of EV71 recombination, we found a more rigid and longer term association of specific genogroups with individual 3Dpol clades; thus genogroups B1, B2, B3 and B4/B5 were associated with RF-E, -D, -G and -A, respectively, whilst C1, C2, C3, C4 and C5 were predominantly associated with RF-W, -V, -L and -T (fig. 1 in McWilliam Leitch *et al.*, 2012). These longer term linkages in EV71 contrasted with much more frequent recombination events amongst species A serotypes, CAV2, CAV4 and CAV10 (Lukashev *et al.*, 2014). Although we lack sufficient sample numbers to estimate recombination half-lives of CVA6, the occurrence of numerous recombination events in the RF-A clade within the past 10 years (Fig. 4) demonstrates rates comparable with other EV-A serotypes.

These inter-serotype recombination rates contrast with the low frequency reported for CAV16. In the analysis of available complete-genome sequences largely originating from China and elsewhere in East Asia, all variants (except the prototype isolate, G-10 isolated in 1953) grouped together throughout the NS region (Fig. 2a) and formed a single recombination group in the 3Dpol region (Fig. S1). The only exception was the sequence of GenBank accession number EU812514, ostensibly isolated in China in 2008, but which was 99.8 % identical in sequence to the prototype G-10 and is a likely laboratory contaminant or a laboratory acquired infection. Further CAV16 sequences characterized from Russia (*n* = 8) and Ukraine (*n* = 1) also fell into this group (Lukashev *et al.*, 2014). Whilst this particular recombination group of CAV16 has clearly persisted for many years and is geographically widespread, analysis of the existing sequences dataset of other CAV16 variants (isolated in Finland, South Africa, Netherlands, Spain) demonstrates the presence of five further recombination groups distinct from the main group (McWilliam Leitch *et al.*, 2012). Whilst limited geographical sampling has clearly led to underestimates of the number of recombination groups of CAV16, existing data point towards substantial differences in recombination frequencies between different enteroviruses, even within the same species, for reasons that remain unclear (Lukashev *et al.*, 2014).

The temporal reconstruction of the recent evolutionary history of CVA6 allowed the timing of recombination events to be, at least in part, reconstructed. This analysis was naturally limited by the available sampling, and clearly the origins of RF-B, -C and -D that possess more divergent VP1 sequence cannot be satisfactorily reconstructed at present. However, for viruses within the main VP1 clade, the most parsimonious interpretation of the distribution of recombination groups was that RF-A was the ancestral form, and that RF-E, -F, -G and -H have emerged more recently following separate recombination events at various times in the past 5–10 years. Being able to infer directionality greatly assists the reconstruction of the actual recombination events that generated these more recently appearing CVA6 variants (Fig. 6). Whilst RF-F and -H appear to have originated through single recombination events with breakpoints in the 2A/2B region (Fig. 6), two or more events could be inferred for RF-G and -E. The position of one of the breakpoints in RF-E within the VP3 gene appears to contrast with previous analyses that have documented the rarity of recombination events between serotypes in the structural gene region (Cuervo *et al.*, 2001; Oberste *et al.*, 2004b; Simmonds, 2006). However, capsid gene sequences either side of the breakpoint are both of CVA6 and the recombination events likely represent a further example of an intra-serotype recombination that has been documented in other enteroviruses (Costa-Mattioli *et al.*, 2003; Oberste *et al.*, 2004b).

The reconstruction of recombination events also led to a number of interesting reinterpretations about CVA6 disease associations. Most importantly, if the evolutionary history of RF-A extends back to before 2000, as implied by the most parsimonious interpretation of the temporal phylogeny (Fig. 4), then it is unexpected that no cases of aHFMD earlier than those first described in 2008 (Österback *et al.*, 2009) have been recorded. It is possible that RF-A infections were initially rare, remaining undetected or not sufficiently characterized clinically until their distribution increased to a point where the change in disease presentations was recognized. Alternatively, RF-A may have originated and spread in a geographical region with limited clinical/virological surveillance and where changes in clinical presentation of CVA6 infections were unrecorded. The other possibility is that the more divergent 2010 isolate, GenBank accession number JQ946053, had independently acquired an RF-A-like NS structural gene; this would push forward the common ancestor of the main group of RF-A variants to 2004, more consistent with its clinical emergence. Such independent acquisition seems similarly likely for the 2003 isolate, GenBank accession number AB779616, that groups closest with the RF-B variant, GenBank accession number AB779614, in VP1 (Figs 1a and 5).

There is a similar possible reinterpretation of the evolutionary age of RF-H, as evidently viruses with NS region sequences matching this recombination group must have existed before 2013 to account for their presence in the evolutionary history of RF-G (through a likely second recombination event). Again, the non-detection of RF-H prior to 2013 may reflect its rarity and lack of surveillance rather than non-pathogenicity. Clearly, more extensive sampling and assignment of recombination groups to a wider range of CVA6 variants is necessary to understand more about the emergence of this and other CVA6 recombination groups.

### Viral determinants of CVA6 clinical phenotypes

There are few, if any, models in which potential differences in disease association between CVA6 variants (or those of any other serotype) can be investigated experimentally. However, some limited conclusions can be drawn by comparing sequence relationships of CVA6 variants associated with differing clinical outcomes. Based on partial VP1 sequences, most studies have found evidence for often distinct clusters of variants associated with aHFMD, although comparisons of sequences from different studies demonstrate little consistent grouping of aHFMD-associated cases between different geographical regions (Chen *et al.*, 2012; Chung *et al.*, 2013; Di *et al.*, 2014; Huang *et al.*, 2013; Lu *et al.*, 2012; Montes *et al.*, 2013; Österback *et al.*, 2009; Puenpa *et al.*, 2014). These comparisons have been problematic, with often only extremely short VP1 partial sequences being available. It was also difficult, a priori, to know whether differences in VP1 sequences are related to the phenotypic change in CVA6 or whether changes elsewhere in the genome are responsible, such as the 5′ UTR or replication-associated genes in the NS region. This difficulty is heightened by the occurrence of recombination in CVA6 genomes that breaks linkages between VP1 lineage with mutations elsewhere in the genome that may influence viral phenotype.

However, by comparing sequences in other parts of the genome (5′ UTR, VP4 and 3Dpol), it was possible to make some inferences over which genome regions are most closely associated with clinical phenotypes. Despite the variability of VP1 sequences, all variants associated with aHFMD after 2008 characterized to date worldwide fell into RF-A, including most Edinburgh samples collected before 2014. Cases of eczema herpeticum reported from Edinburgh in 2014 (Sinclair *et al.*, 2014) fell into a novel clade, RF-H. The significant differences between recombination group and clinical presentations (Table 1) were supported by individual observational studies. Taiwanese cases associated with herpangina were consistently RF-E (GenBank accession numbers JQ94600–JQ94602), whilst those collected in 2010–2011 and associated with HFMD (GenBank accession numbers JQ94603–JQ94605 and JN582001) were RF-A. A differentiation of HFMD-associated variants in Japan from those collected pre-outbreak was similarly evident. Although lacking 3Dpol sequences, CVA6 variants from 2011 that were not associated with HFMD possessed VP1 sequences closely similar to RF-D and -C (GenBank accession numbers AB779617 and JQ964234, respectively). In contrast to the association of RF-A and -H with aHFMD, no associations between clinical presentations and 5′ UTR or VP4 region phylogenies were apparent.

Whether the RF-H variants detected in Edinburgh in 2014 are more geographically widespread requires further genetic characterization of NS gene regions of CVA6 isolates from other geographical regions; recombination group cannot be reliably predicted from VP1 phylogenies. However, none of the published VP1 sequences fall into the clade formed by recent Edinburgh variants in this genome region (Fig. 1a), suggesting a current, relatively limited geographical distribution. Further work is clearly required to better characterize any possible clinical or epidemiological differences between RF-A and RF-H CVA6 variants and determine whether or when RF-H replaces RF-A globally. This might be performed by the consortium of clinical laboratories in Europe, Asia and Australia that previously documented the recombination dynamics of other enteroviruses (Cabrerizo *et al.*, 2014; McWilliam Leitch *et al.*, 2009, 2010, 2012). Further samples and surveillance data are potentially available from larger reference laboratories, such as the US and European communicable disease centres. Such studies may contribute to a better understanding of the driving forces behind such replacement processes, such as the appearance of RF-A in 2008, and the numerous examples amongst EV-B serotypes, and their relationships with clinical disease and herd immunity. These are crucial for gaining a better understanding of the pathogenesis of enterovirus infections and their variability in clinical phenotypes.

## Methods

### 

#### Clinical samples.

Twelve **s**amples were obtained from patients presenting with eczema herpeticum as described previously (Sinclair *et al.*, 2014), along with other samples from Edinburgh patients with aHFMD infected with CVA6 (Harvala *et al.*, 2014). Fifteen samples from a previous CVA6 outbreak in Finland (Österback *et al.*, 2009) were also analysed.

#### PCR and nucleotide sequencing.

Samples of vesicle fluids (from eczema herpeticum/aHFMD cases) or cerebrospinal fluid were extracted using a QIAamp MinElute Virus Spin kit (Qiagen) according to manufacturer’s instructions. A one-step reverse transcription/first-round PCR was undertaken. Each 20 µl reaction contained 10 µl SuperScript III One-Step RT-PCR 2× Master Mix (Invitrogen), 0.8 µl SuperScript III RT/Platinum *Taq* High Fidelity Enzyme Mix (Invitrogen), 1 µl each forward and reverse outer primers for VP4, VP1, 3Dpol or 5′ UTR region amplification (Table 2), and 6 µl nucleic acid. Cycling conditions for VP1 amplification were 43 °C for 60 min, then 20 cycles of 53 °C for 1 min and 55 °C for 1 min, one 70 °C hold for 15 min, one 94 °C hold for 2 min, then 40 cycles of 94 °C for 30 s, 45 °C for 30 s and 68 °C for 1 min 45 s, followed by a 68 °C hold for 5 min. Cycling conditions for VP4, 3Dpol and 5′ UTR were slightly modified so that the 45 °C annealing temperature was raised to 50 °C, as described previously (Leitch *et al.*, 2009). The 20 µl second-round nested PCR contained 4 µl reaction buffer (Promega), 0.2 µl dNTPs, 1 µl each forward and reverse VP1 or VP4 inner primers, 0.1 µl *Taq* polymerase (Promega) and 1 µl first-round product. CVA6-specific PCR used VP1-specific primers (Table 2).

**Table 2.** Primers used for enterovirus genome amplification by nested PCR

Virus	Region	Primer	Sequence (5′→3′)
CVA6	VP1	2347s	GARGCTAACATYATAGCTCTTGGAGC
CVA6	VP1	2407s	GACACYGAYGARATYCAACAAACAGC
CVA6	VP1	3296a	CGRTCRGTTGCAGTGTTWGTTATTGT
CVA6	VP1	3326a	CCYTCATARTCHGTGGTGGTTATGCT
Enterovirus	VP4	458s	CCGGCCCCTGAATGYGGCTAA
Enterovirus	VP4	547s	ACCRACTACTTTGGGTGTCCGTG
Enterovirus	VP4	1087a	TCWGGHARYTTCCAMCACCANCC
Enterovirus	VP4	1125a	ACATRTTYTSNCCAAANAYDCCCAT
Enterovirus A	VP1	2268s	CCNTGGATHAGYAACACNCAYT
Enterovirus A	VP1	2332s	TNASNATYTGGTAYCARACNAAYT
Enterovirus A	VP1	3016a	GANGGRTTNGTNGKNGTYTGCCA
Enterovirus A	VP1	3109a	GGRTANCCRTCRTARAACCAYTG
Enterovirus	5′ UTR	178s	HCAAGYACTTCTGTYWCCCCSG
Enterovirus	5′ UTR	370s	GGCTGCGYTGGCGGCCTRC
Enterovirus	5′ UTR	477a	TTAGCCRCATTCAGGGGCCGG
Enterovirus	5′ UTR	573a	RGAAACACGGACACCCAAAGTAGT
Enterovirus	3Dpol	OS	GGBGGNACHCCHACNAARMGVATGCT
Enterovirus	3Dpol	IS	AARAGAATGCTYATGTAYAAYTTYCC
Enterovirus	3Dpol	IAS	ARDCCRTAYTTRTCCATRCAYTCYTT
Enterovirus	3Dpol	OAS	TCRTCYTTBACRTADGTYACCATTGG

#### Complete genome sequencing.

Complete genome sequencing of 15 CVA6 strains was carried out at the MRC University of Glasgow Centre for Virus Research using a metagenomic approach on the Illumina MiSeq platform. Extracted RNA was converted to double-stranded cDNA using a Maxima First Strand cDNA Synthesis kit (Thermo Scientific) and a Second Strand cDNA Synthesis kit (NEB). Library preparation was carried out using Nextera XT (Illumina). Alignment files and consensus sequences were generated using Tanoti (http://www.bioinformatics.cvr.ac.uk/Tanoti/index.php).

#### Sequence alignment and strain analysis.

Forward and reverse sequence reads were reconciled and aligned in the sse 2.1 sequence editor package (www.virus-evolution.org) (Simmonds, 2012). Datasets included all available complete genome sequences of EV-A types, although due to their number, EV71 sequences were excluded if <3 % divergent over the genome from other EV71 variants. Phylogenetic trees were reconstructed by the maximum-likelihood method using optimal nucleotide substitution models identified in ModelTest as implemented in the mega6 package (Tamura *et al.*, 2013). Recombination analysis used the programs Sequence Distance and Grouping Scan in the sse package (Simmonds, 2012).

All sequences obtained in the study, along with geographical and date information on published sequences included in the analysis, are listed in Table S1.

A Bayesian MCMC method implemented in beast 1.74 (Drummond *et al.*, 2006) was used to estimate temporal phylogenies and rates of evolution (Drummond & Rambaut, 2007). VP1 region sequences were analysed using constant and exponential population sizes as priors with a chain length of 50 million and a relaxed log-normal molecular clock model that allowed evolutionary rates to vary between lineages. All other parameters were optimized during the burn-in period. Substitution rates and phylogenies were comparable between outputs generated by the two different priors; the results from exponential population sizes were used for the analysis. Output from beast was analysed using the program tracer (http://beast.bio.ed.ac.uk/Tracer) and through construction of a maximum clade credibility tree using TreeAnnotator/FigTree.
